# Selection and prediction of metro station sites based on spatial data and random forest: a study of Lanzhou, China

**DOI:** 10.1038/s41598-023-49877-6

**Published:** 2023-12-18

**Authors:** Quanfu Niu, Gang Wang, Bo Liu, Ruizhen Zhang, Jiaojiao Lei, Hao Wang, Mingzhi Liu

**Affiliations:** 1 School of Civil Engineering, Lanzhou University of Technology, Lanzhou, 730050 China; 2 Emergency Mapping Engineering Research Center of Gansu Province, Lanzhou, 730050 China; 3 Academician Expert Workstation of Gansu Dayu Jiu Zhou Space Information Technology Co., Ltd, Lanzhou, 730050 China

**Keywords:** Environmental social sciences, Engineering

## Abstract

Urban economic development, congestion relief, and traffic efficiency are all greatly impacted by the thoughtful planning of urban metro station layout. with the urban area of Lanzhou as an example, the suitability of the station locations of the built metro stations of the rail transit lines 1 and 2 in the study area have been evaluated using multi-source heterogeneous spatial data through data collection, feature matrix construction, the use of random forest and K-fold cross-validation, among other methods. The average Gini reduction value was used to examine the contribution rate of each feature indicator based on the examination of model truthfulness. According to the study's findings: (1) K-fold cross-validation was applied to test the random forest model that was built using the built metro stations and particular factors. The average accuracy of the tests and out-of-bag data (OOB) of tenfold cross-validation were 89.62% and 91.285%, respectively. Additionally, the AUC area under the ROC curve was 0.9823, indicating that this time, from the perspective of the natural environment, traffic location, and social factors The 19 elements selected from the views of the urban function structure, social economics, and natural environment are closely associated to the locations of the metro station in the research region, and the prediction the findings are more reliable; (2) It becomes apparent that more than half of the built station sites display excellent agreement with the predicted sites in terms of geographical location by superimposing the built metro station sites with the prediction results and tally up their cumulative prediction probability values within the 300 m buffering zone; (3) Based on the contribution rate of each indicator to the model, transport facilities, companies, population density, night lighting, science, education and culture, residential communities, and road network density are identified as the primary influential factors, each accounting for over 6.6%. Subsequently, land use, elevation, and slope are found to have relatively lower contributions. The results of the research provided important information for the local metro's best location selection and planning.

## Introduction

Given the explosive growth of the Chinese economy, urban rail transit has been instrumental in improving the effectiveness of urban public transportation, reducing congestion, optimizing the use of readily available space, and improving the living environment^1^. To date, approximately 53 cities in China have opened and operated urban rail transit, of which the selection of urban metro station sites needed to be done in conjunction with the spatial layout of the city, work commuting and the layout of tourist attractions^2^. The reasonable design of metro stations contributes significantly to the economic growth of cities, the reduction of commute times, the growth of tourism, and the name-card impact of the city.

There are often the following categories for the site selection issue. One is the traditional site selection methods, which is based on the development of various industries within the proposed site selection area, combined with the proposed site and surrounding environment to determine the site selection. This method is easily influenced by subjective factors of the executors^3^. The second is to use mathematical modeling methods, which primarily rely on specific algorithms and multivariate analysis to establish a location model and are more popular due to their better siting accuracy^3^. The third is to use the geo-information system (GIS) technique, which is based on the spatial data with geographical analysis and mathematical modeling to satisfy the demands of the site selection plan^3^. For example, by incorporating the spatial lag model (SLM) and ordinary least squares (OLS) models, Wang et al. employed buffer zone analysis and spatial autocorrelation to explore the spatial layout features and the affecting factors for of Guangzhou commercial gyms, it is concluded that population density and education level were the most significant factors^4^. Wang et al.^5^ used various spatial analysis methods to analyze the characteristics of spatial location differences between traditional retail and new retail in Shanghai and used spatial econometric models to explore the differences at the level of location factors. Wang et al.^6^ used a multi-objective optimization algorithm to solve the problem based on data such as the coverage rate of the firefighting responsibility area to provide a reference for fire station locations. In recent years, other methods, such as hierarchical analysis^7^ and game analysis^8^ have been applied to site selection analysis. These methods mostly analyze the influence degree of various factors on site selection from the perspective of spatial analysis and statistical methods from the macro level to obtain the comprehensive scores of candidate sites^9^.

With the continuous development of computers, big data and smart city technologies, the emergence of multiple sources of geospatial data provides an important data source for site selection problems. At the same time, machine learning algorithms are becoming increasingly popular in site selection research. As a result, an increasing number of scholars have started to use multisource spatial data as input to build fitted model using machine learning algorithms for site selection research. For example, Deng et al. used a multi-model fusion algorithm to build a bank branch location model based on multisource data, showing that this method has higher accuracy than the traditional method^10^. Based on Point Of Interest (POI) data, Yang et al.^11^ used various spatial analysis methods and combined ordinary least squares (OLS), spatial lag model (SLM) and spatial error model (SEM) models to verify that public service facilities have a significant positive effect on the surrounding space. Jin et al.^12^ used machine learning methods to analyze the importance of different influencing factors on the location of new retail shops. Zhang et al.^13^ used multisource spatial data with geographical and commercial characteristics to construct a feature matrix, used the random forest algorithm to construct a fitting model for predicting the conversion rate of shop visits, and finally obtained the spatial distribution of the suitability of advertising locations. Wang et al.^14^ obtained POI data and basic data from the population census, combined these data with the existing urban area by obtaining POI data and population census base data, and further combined the data with the existing spatial layout of elderly facilities in Wuhan city and decision tree model, and conducted a quantitative simulation of the distribution pattern of elderly facility location selection, concluding that the method could avoid the subjectivity of planning location selection as much as possible. Huang et al.^15^ used POI data and other bases, fused multisource heterogeneous spatial data and a random forest model to analyze and evaluate the suitability of the Changsha Sexy Tea Store layout, and concluded that the contribution of competitive environment factors to the model was greater. The above multisource spatial data and machine learning algorithms enrich the theoretical study of site selection while providing a reference for such a selection.

For the selection of metro station, it is undoubtedly crucial to comprehensively consider multiple influencing factors and appropriate algorithms. However, different algorithms have their own application environment and limitations. For example, hierarchical linear regression is applied to fuzzy group decision-making^16^, and it is easy to have wrong regression results, leading to instability and reliability of the model in the face of complex nonlinear models, Outliers and multiple covariates^17^. Multilayer perceptron (MLP), as a common neural network model, has strong nonlinear modeling ability^18^, but there are also some problems that need to be overcome, such as overfitting risk, hyperparameter selection, local optimal solution, computational complexity, and black box model^19^. In recent years, graph neural networks are mainly used to process graph-structured data^20^, however, more complex processing and techniques such as adjusting weights may be required in unbalanced datasets^21^. By comparison, it is found that random forest algorithm has the advantages of simple operation, easy implementation, small amount of calculation, and shows very amazing performance in classification and regression.

The construction of rail transit line in Lanzhou plays an important role in improving the efficiency of urban traffic and relieving congestion. However, as a typical river valley city, the Yellow River flows from west to east through the city, and the urban areas are spread out like pearls on both sides of the Yellow River. So, a single rail transit line from east to west can meet the needs of most of the peers in the city. Therefore, for the construction of rail transit line in such cities, it is particularly important to select reasonable metro station sites. Our study uses multisource heterogeneous spatial data, combining with random forest algorithm and spatial analysis methods to 1) explore the methods for the selection of metro station from various factors, 2) evaluate the suitability of the built metro station sites, 3) provide reference for other rail transit route planning and station site selection in the similar region.

## Data sources and methods

### Research area overview

**Figure 1:**
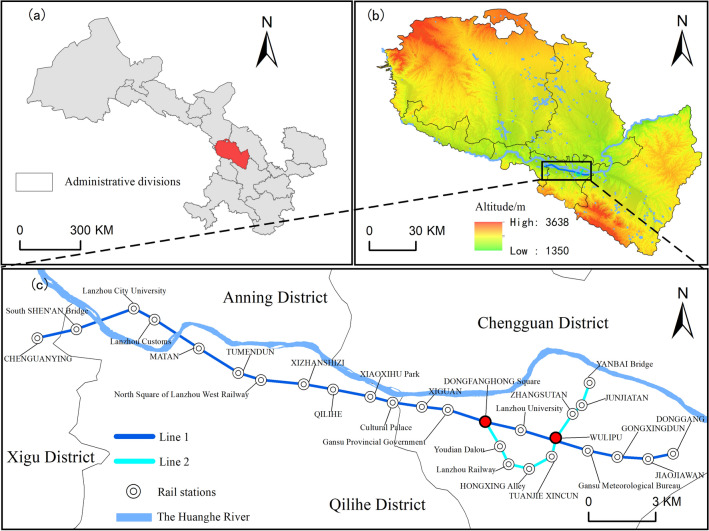
Spatial distribution of the study area and metro stations [(**a**) administrative division of Gansu Province, (**b**) Lanzhou city, (**c**) Lanzhou Metro Lines 1 and 2].

Lanzhou, the capital city of Gansu Province, is located at the geometric center of China's map. It is not only an important center city, industrial base, and comprehensive transportation hub in the northwest region of China, but also a vital node along the Silk Road Economic Belt^22^. Due to its distinctive terrain and topography, the Yellow River flows from west to east through the city. Between the two mountains in the north and south, the urban area is distributed like a string of beads on both sides of the Yellow River. Narrow from north to south and longer from east to west, it is a typical valley shaped and strip shaped city. Based on topographical factors, the main urban area is densely populated and very congested. In order to alleviate traffic pressure, increase the efficiency of public transport and improve the environment, Lanzhou has completed the first phase of the east–west Transit Railway No. 1 and the second phase of the Transit Railway No. 2 project (Fig. 1). To improve utilization, Lanzhou metro Line 1 is the first subway line in China to cross the Yellow River twice. For rail transit systems designed for such urban terrain characteristics, it is particularly important to reasonably set up subway station locations to facilitate people's lives, improve traffic efficiency, and relieve congestion.

### Data source and preprocessing

The data used in this study and their attributes are shown in Table 1 below:

**Table 1:** Data name and attribute information.

Name	Time	Source	Introduction
POI	2022	Amap (https://bs.amap.com/)	39,819 POI data were obtained by calling the Amap(https://bs.amap.com/) API via web crawler
Population data	2022	Open Spatial Demographic Data and Research (http://www.worldpop.org)	Spatial resolution of 3 radians
Nighttime light data	2021	luojia1-01 (http://59.175.109.173:8888/app/login.htm1)	Night lighting data is highly correlated with secondary and tertiary Gross Domestic Product (GDP)
Digital Elevation Model (DEM)	2019	NASAEARTH DATA (https://earthdatanasa.gov/)	The spatial resolution is 30 m, which is a good reflection of the elevation of the study area
Data on land use	2020	Data Sharing Services System (http://data.casearth.cn/sdo/detail/5fbc7904819aeclea2dd7061)	Used to reflect surface cover condition
Road network data	2022	OpenStreetMap(https://www.openstreetmap.org)	Timely updating and easy access, often used to analyze urban traffic conditions
Administrative division data	2022	Data Sharing Services System (http://data.casearth.cn/sdo/detail/5fbc7904819aeclea2dd7061)	Reflects administrative boundaries of districts

#### POI data

**Figure 2:**
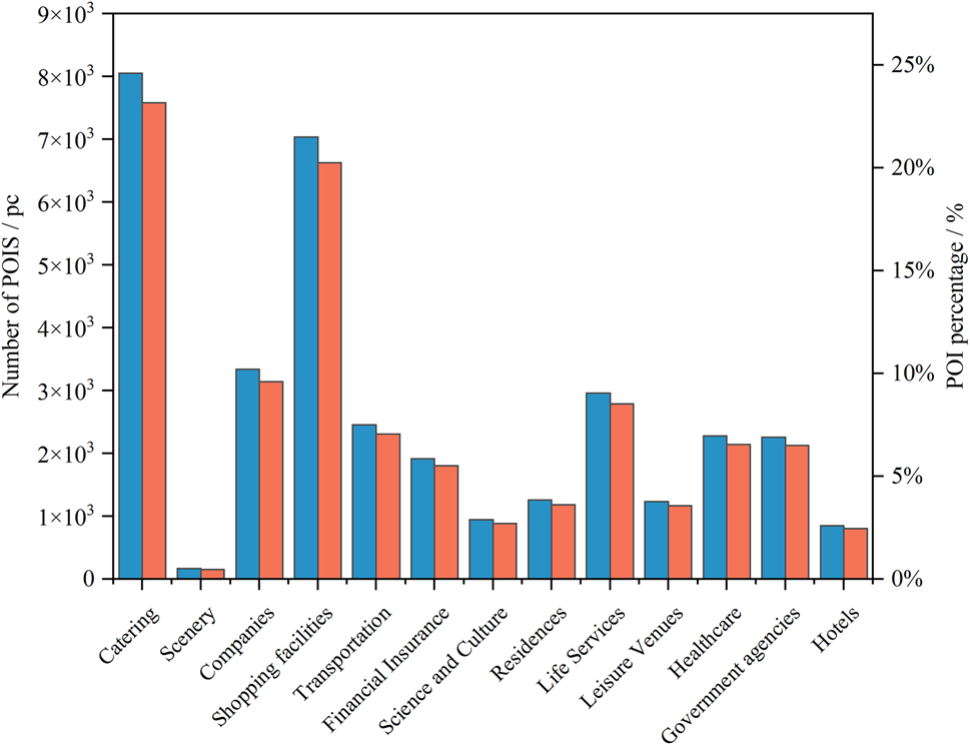
Number and percentage of POI.

In this study, we obtained POI data (Table 1) by calling the web-side open API provided by Amap (https://bs.amap.com/) through a web crawler. Based on Amap's classification standard, these data were classified into 13 categories, such as science, education, culture, living, finance, insurance, and so on (Fig. 2). To analyze the rationality of the layout of existing station sites, these data were processed as follows: Firstly, we calculated and normalize the kernel density of every category^23,24^ with Eqs. (1) and (2). Secondly, these kernel densities were projected and resampled to 30 m^25^. Finally, the feature matrix was constructed to facilitate the spatial analysis of the station sites. The kernel density analysis algorithm used in this study is as follows:

1 $$D=\frac{3{\left(1-{scale}^{2}\right)}^{2}}{\pi {r}^{2}}$$

where $r$ denotes the search radius, and ${\text{scale}}$ is the ratio of the distance from the center of the grid to the point or line object to the search radius^26^.

The normalization function is:

2 $${Value}_{norm}=\frac{Value-{Value}_{min}}{{Value}_{max}-{value}_{min}}$$

when ${Value}_{norm}$ represents the normalized pixel value, Value represents the pixel value before normalization, ${Value}_{max}$ represents the maximum pixel value, and ${Value}_{min}$ represents the minimum pixel value.

#### Socioeconomic data

Socioeconomic data include population as well as night light data. Among them, the World Pop Global High Resolution Population Programme dataset (http://www.worldpop.org) provides population data with a spatial resolution of 3 radians^27^. For ease of application, the population data are first raster projected and the spatial resolution is resampled to 30 m for constructing the feature dataset; the nighttime lighting data are derived from the Luoga 1 satellite data launched by Wuhan University satellite data^28^; due to the high correlation between nighttime lighting data and the Gross Domestic Product(GDP)of the second and third industries^29^,this study uses such data to reflect the nighttime human flow data. The following equations are mainly used for radiometric calibration of nighttime lighting data:

3 $$L={DN}^{3/2}\times {10}^{-10}$$

where ${\text{L}}$ represents the radiance value after absolute radiometric correction, and ${\text{DN}}$ represents its grayscale value.

#### Terrain and land use data

The topographic data are mainly digital elevation model (DEM) with a spatial resolution of 30 m, which can well reflect the elevation of the study area. In this study, ArcGIS was used to extract the topographic slope to construct the spatial dataset of site selection features. The land use data are mainly used to reflect the surface cover condition^30^.

#### Road network data

**Figure 3:**
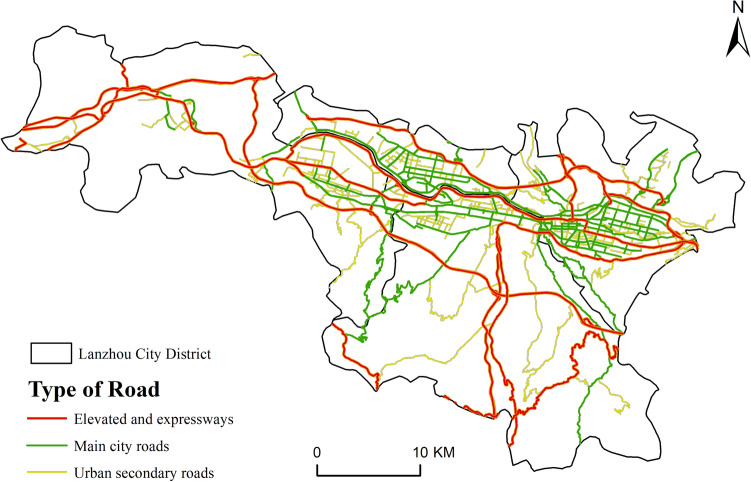
Road network distribution in Lanzhou city.

The road network data used in this study are up-to-date and easily accessible, and are often used to analyze urban traffic conditions. For ease of application, these data were cleaned before use (Fig. 3), and then their weighted road network density (which is a kernel density analysis of roads of different levels, after which a weight is given to each road in terms of importance, and finally the kernel densities are superimposed together)^31^ was calculated for the construction of the siting feature spatial dataset.

### Research methodology

**Figure 4:**
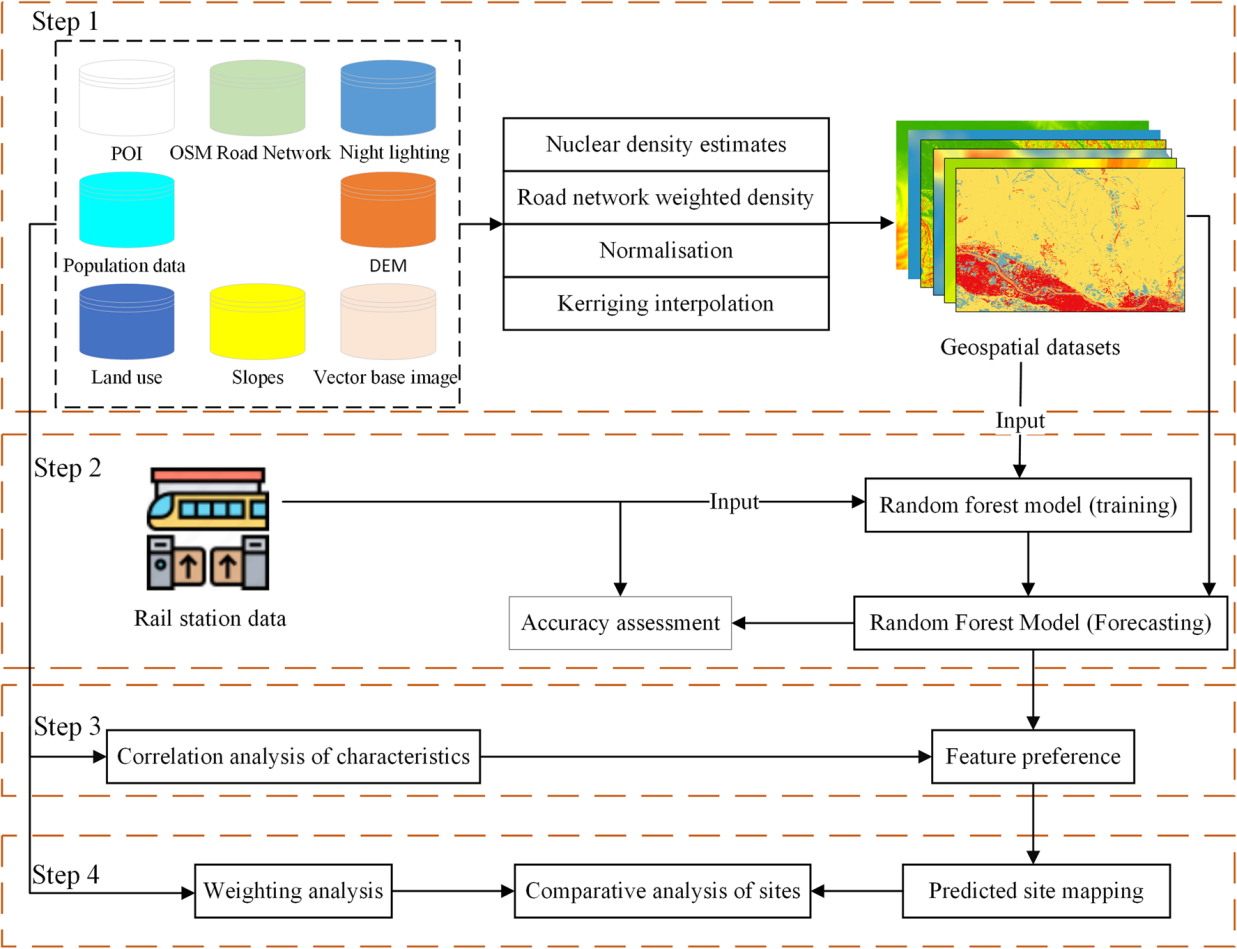
Technical flow.

Gridding the study area can provide a unified scale for spatial comparative analysis and can also simplify the merging and separation of multisource spatial data and improve the efficiency of data processing. Therefore, in this study, based on the reference of Huang Qin et al.^15^ and Gong^32^, combined with the reality of the study area, the study area was gridded with a grid of 100 m × 100 m. First, preprocessing operations such as cleaning and projection are carried out on various data. Then, kernel density, weighted density, and kriging interpolation (which is a statistically based interpolation method, also known as spatial local interpolation) are computed on different data^33^, Then, normalization is carried out to construct the feature dataset. Finally, the dataset is substituted into the random forest model to train the prediction, and the result obtained is compared and analyzed with the original result. The flow of the research method in this paper is as follows (Fig. 4).

#### Random forest model

The random forest model is a type of ensemble model that consists of multiple decision trees^34^. It has been widely used in remote sensing classification for solving multidimensional nonlinear classification problems due to its advantage in spatial fitting accuracy^35–37^. The random forest model combines the bagging algorithm and random feature selection. During the learning process, N decision trees are constructed and the prediction results of each tree in the forest are statistically counted when fitting a sample for regression. The best result is then selected through voting. The random forest has two random features: random selection of features and random selection of samples.

#### Constructing the feature matrix

The process of constructing the feature matrix is as follows: first, 19 features affecting the layout of metro stations in the study area are selected, and the feature matrix is constructed by calculating the corresponding feature values in each grid; second, the POI data of 27 completed metro stations are crawled and their corresponding feature values are extracted using the dichotomy method to construct the training dataset; then, 70% of the feature dataset is randomly selected as the training data, and the remaining 30% together with the out-of-bag data (OOB) are used as the validation data; finally, the completed feature matrix is input into the pretrained random forest model for regression operations. The remaining 30%, together with the out-of-bag (OOB) data, are used as validation data. Finally, the completed feature matrix is fed into a pretrained random forest model for regression to obtain the probability distribution of the underground station locations on each grid.

**Figure 5:**
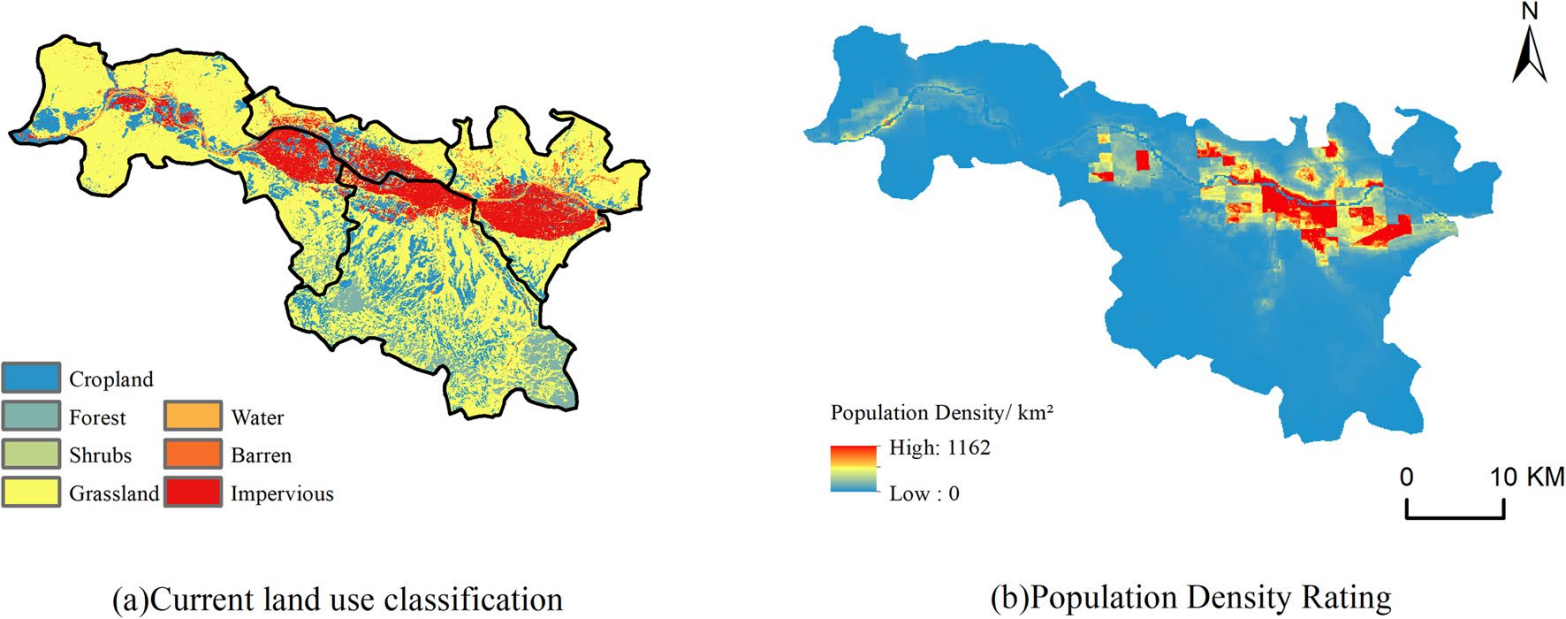
Distribution of land use types and population density in the study area.

**Figure 6:**
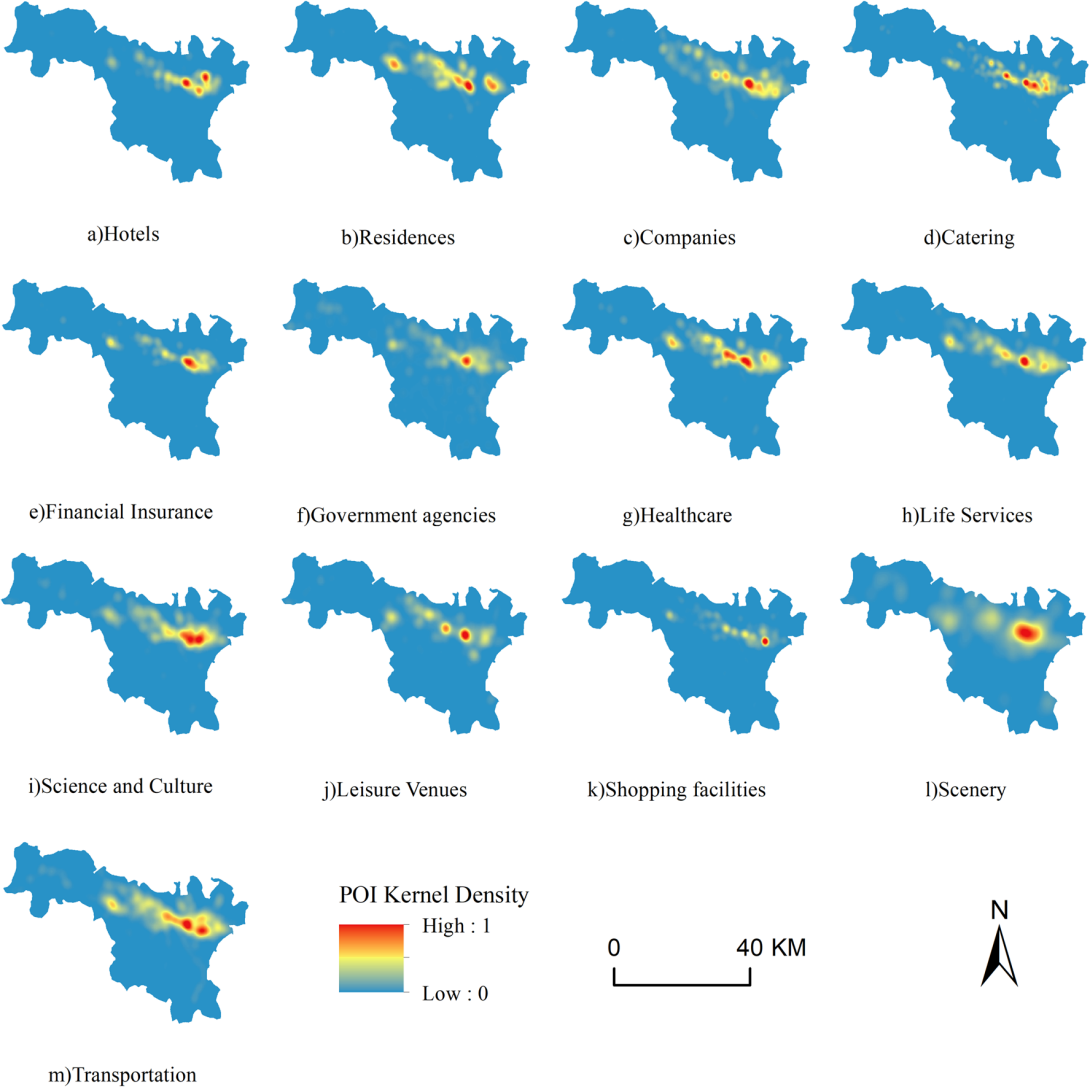
Kernel density distribution of POIs.

Through field investigation and overlaying analysis with various types of POI data, the metro stations are preferably located in places with relatively high pedestrian flow, such as office buildings, schools, residential areas, pedestrian streets and large shopping malls. In this experiment, with reference to the study by Guo et al.^38^, the characteristic factors related to the location of metro stations were selected from four categories: natural environment, transportation location, socioeconomic and urban functional structure. And a total of 19 characteristic factors from the four categories were constructed, namely density of food and beverage service facilities, Digital Elevation Model (DEM), slope, road network density, density of living service facilities, density of pedestrian flow, population density, density of land use data, density of scenic services, density of corporate services, density of shopping services, density of transportation services, density of financial and insurance services, density of scientific, educational and cultural services, density of residential communities, density of sports and leisure services, density of health care, density of government agency services and density of accommodation services^39^. Figure 5 shows the spatial distribution of land use types and population density in the study area, while Fig. 6 shows the distribution of other types of POI core density.

#### Performance criteria

Usually, the performance of a model is measured by the kappa coefficient and accuracy. However, this method ignores the posterior probability of the model and cannot fully reflect the model's performance. The area under the ROC curve (AUC value)^40^ can effectively evaluate the performance of the model and measure other indicators such as the posterior probability and sorting performance. Therefore, it has been widely used in model reliability evaluation . Therefore, this paper uses the ROC curve and AUC area value to evaluate the reliability of the established random forest model. The formula for calculating the AUC value is as follows:

4 $$AUC=\frac{{\sum }_{{ins}_{i}\in positiveclass}{rank}_{{ins}_{i}}-M\left(M+1\right)/2}{M\times N}$$

In the formula, $AUC$ is the area under the ROC curve, ${rank}_{{ins}_{i}}$ is the ranking of the probability score of the i-th positive sample, $\sum {ins}_{i}\in positiveclass$ represents the number of positive samples, and M and N represent the numbers of positive and negative samples, respectively.

To reduce the risk of overfitting and improve the generalization ability of the model, we use the K-fold cross-validation method to evaluate the accuracy of the model^41^. Specifically, the sample data are divided into 10 equal parts, ${K}_{1},{K}_{2}$,${K}_{3}$,$\cdots ,{K}_{10}$. One part ${K}_{i}$ is selected as the test dataset, and the remaining 9 parts are used as the training dataset. Then, i groups of training and testing sets {(${\text{Train}}_{i}$, ${\text{Test}}_{i}$), i = 1,2,3,∙∙∙,10} are constructed, and the precision and recall are calculated for each group.

#### Mean decrease gini (MDG)

To determine the importance of each feature (indicator), this study uses the mean decrease Gini (MDG)^42,43^to measure its contribution to the random forest model. The formula for calculating MDG is as follows:

5 $${MDG}_{r}=\frac{{\sum }_{i=1}^{n}{\sum }_{j=1}^{t}{D}_{Grij}}{{\sum }_{r=1}^{m}{\sum }_{i=1}^{n}{\sum }_{j=1}^{t}{D}_{Grij}}$$

where ${MDG}_{r}$ is the importance of the r-th feature among all features; $n$ is the number of decision trees;$t$ is the number of nodes in a single tree;$m$ is the number of all features; and ${D}_{Grij}$ is the Gini index reduction coefficient of the r-th feature at the j-th node of the i-th tree.

## Results

### Model reliability analysis

**Figure 7:**
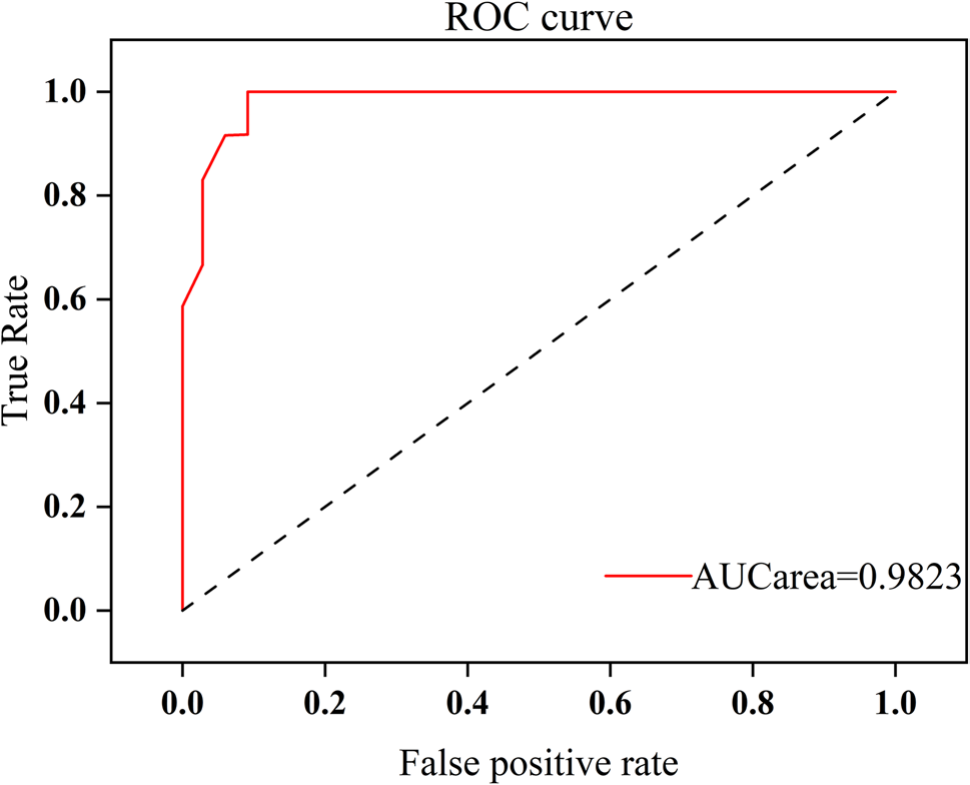
ROC curve and AUC area.

To further verify the reliability of the model, the central location of the completed metro station was taken as the dependent variable, while 19 influencing factors, such as shopping centers, catering services, and so on, were taken as independent variables to construct a fitted model for metro station evaluation based on the random forest model. We gridded the study area at 100 m × 100 m to obtain 347 × 305 grids and preprocessed the corresponding features to create 105,832 sets of samples. Based on the K-fold cross-validation method^44,45^, the samples were divided into 10 equal parts, of which 9 were used as training data and 1 was used as validation data, taking turns for experiments. The results show that the average test accuracy of tenfold cross-validation is 89.62%. The out-of-bag (OOB) data were measured to have a test accuracy of 91.285% in this model constructed, indicating that the model is more desirable. To further analyze the performance of this model, we plotted the ROC curve (Fig. 7), and it can be seen that the ROC curve showed an obvious step-left upward trend, and the AUC area in this state was 0.9823. It can be seen that our model established in this study has good reliability.

### Analysis of driving factors

**Figure 8:**
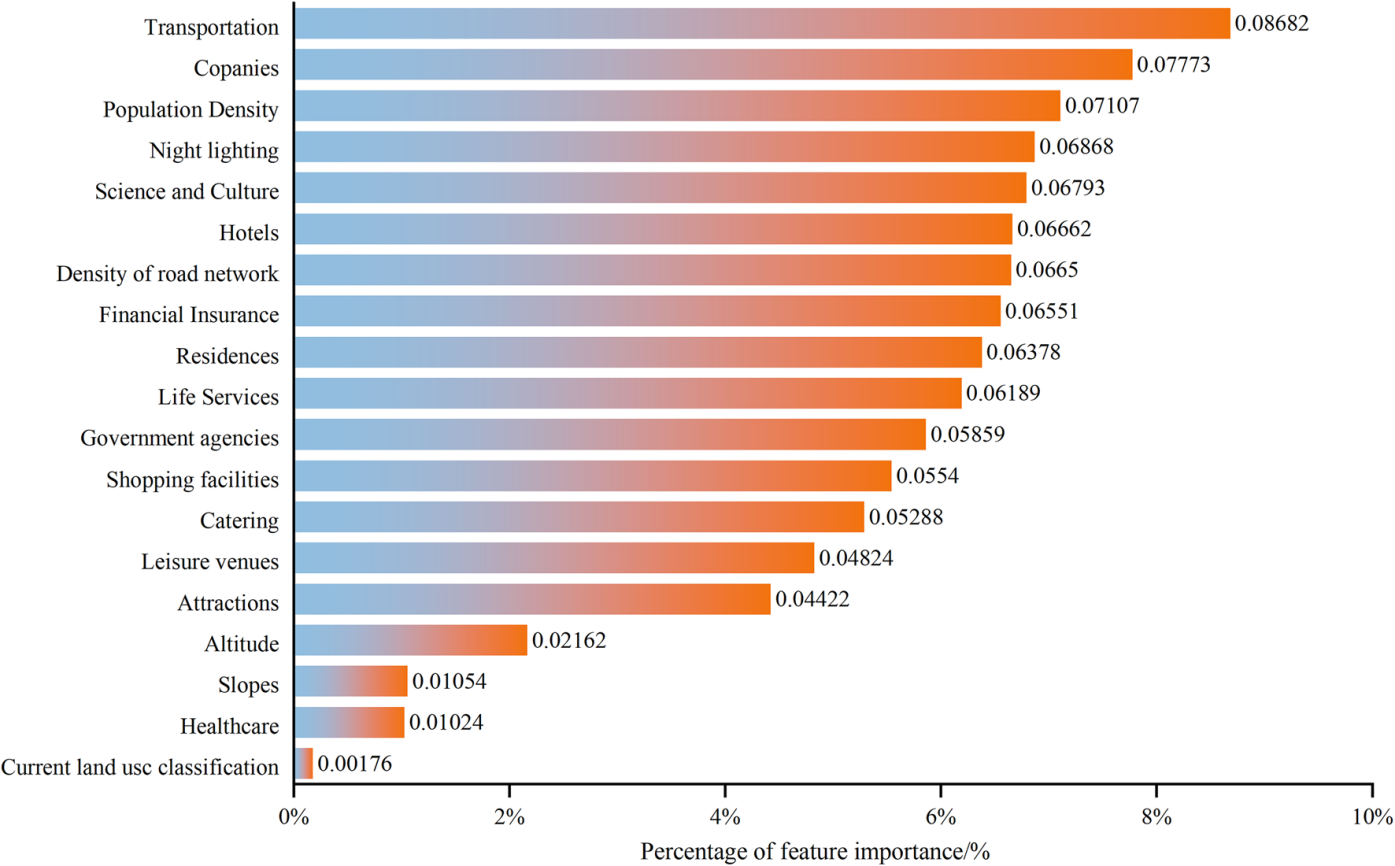
Ranking the importance of every indicator.

To further analyze the factors influencing the selection of subway station locations, we calculated the contribution rate of each influencing factor to the established random forest model using the MDG model (Fig. 8). The ranking of indicator importance revealed that the indicators with a contribution rate greater than 6.6% are, in descending order: transportation facilities, companies and enterprises, population density, night lighting, science and education culture, residential areas, and road network density. Transportation facilities are the most important, accounting for 8.682% of the total, indicating that connecting subway stations with public transportation facilities or planning them near transportation facilities can significantly improve travel convenience and is a necessary condition for selecting locations in commercial centers. Companies and enterprises account for 7.773% and rank second in importance, which is consistent with the spatial distribution results of subway site suitability (the correlation between the predicted site and the constructed site, if the cumulative sum of the predicted probability values of the predicted site falling within the buffer zone of the constructed site is greater, the suitability of the site is considered to be higher, and in the opposite case it is lower). Areas with more businesses typically have higher population density and flow, making them suitable for subway stations that reduce employee commuting time. Population density and night lighting are also important factors, accounting for 7.107% and 6.868%, respectively, and are consistent with the spatial distribution results of subway site suitability. Urban areas with high population density and prosperous business activity have a high concentration of human traffic, making subway stations in these areas a good way to facilitate travel and promote the local economy. Science and education culture is also a significant factor, accounting for 6.793%, indicating a higher demand for subway stations near schools and universities in areas with high population density. Residential areas rank sixth in importance, accounting for 6.662%, which means that subway stations in highly concentrated residential areas are in high demand, connecting residential areas with urban areas to meet the travel needs of more people. Road network density accounts for 6.65% and ranks seventh in importance. Planning subway stations in areas with high road network density can help alleviate traffic congestion and improve road accessibility, which is closely related to commercial and industrial activities. Finally, indicators with lower contribution rates, such as land use, slope, and elevation, account for 0.176%, 1.054%, and 2.162%, respectively. These indicators have relatively low spatial heterogeneity due to the existing subway stations being mainly located in areas with relatively high economic development levels in the city and are mainly built underground.

### Spatial distribution of site suitability

**Figure 9:**
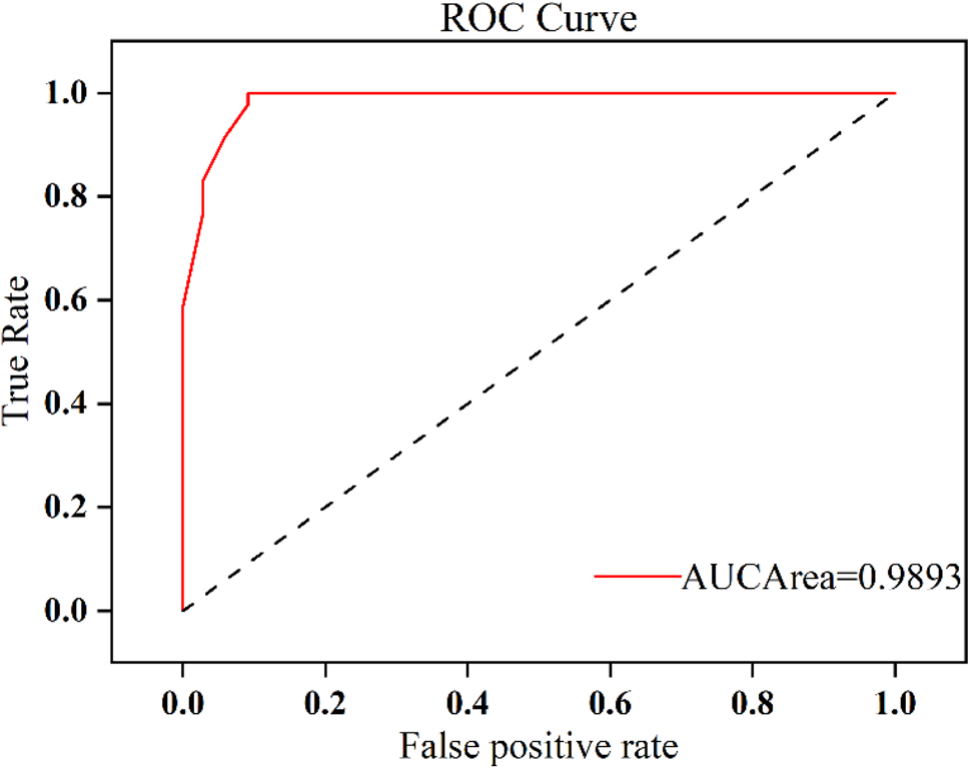
ROC curve and AUC area after model optimization.

**Figure 10:**
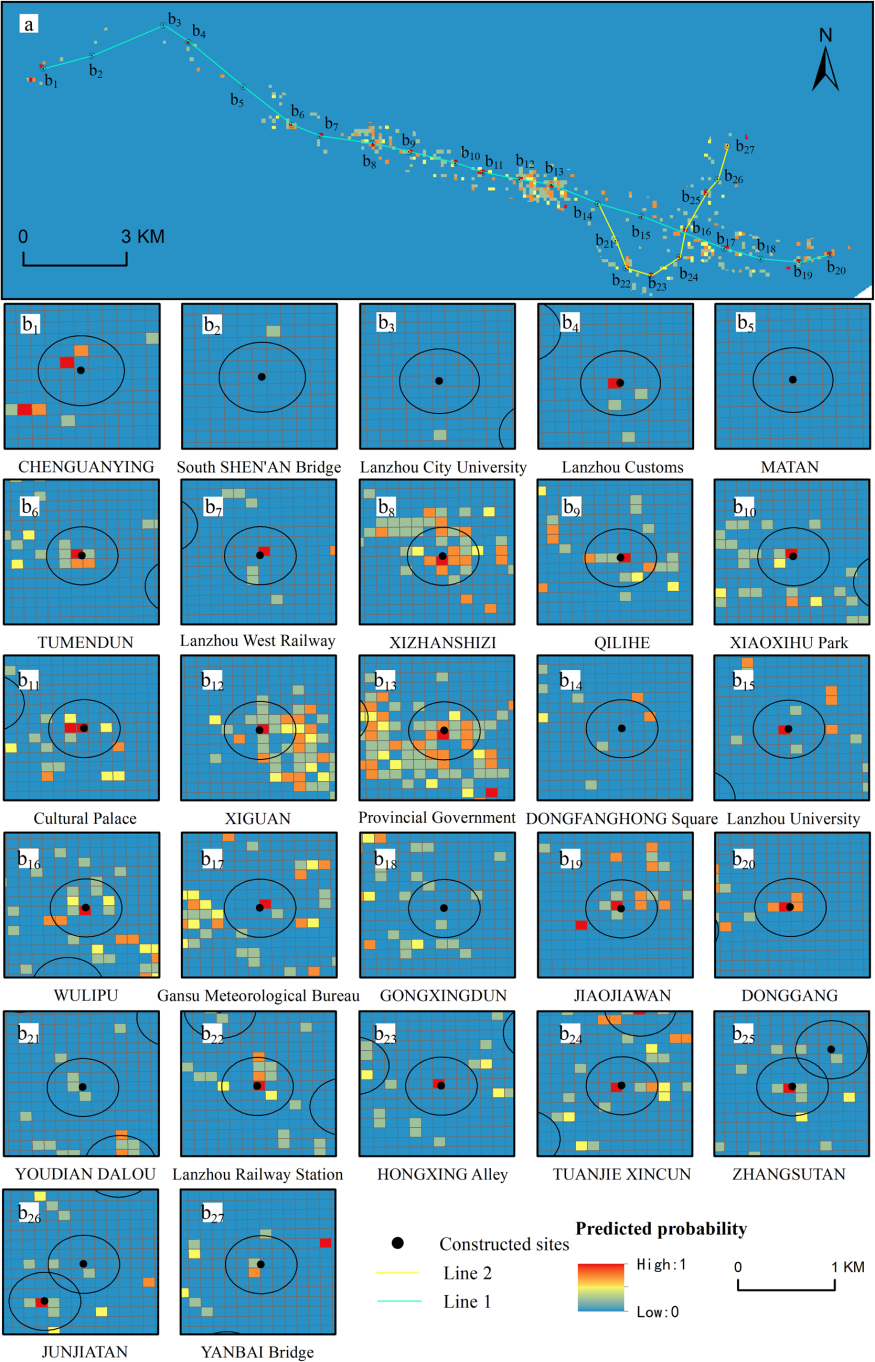
Spatial distribution of the suitability of completed subway station sites ((**a**) The full map of the predicted probability distribution of railway stations (**b**_**i**_) Spatially predicted probability distributions for each rail station).

To improve the accuracy of the model, feature optimization were carried out on various types of features constructed with random forest algorithm. In the end, we removed four features with lower relevance and contribution rankings, i.e., elevation, slope, healthcare and land use. Comparing the results from 3.1, the average detection accuracy of tenfold cross-validation was 89.73%, and the test accuracy of the out-of-the-bag (OOB) data reached 91.357%, with an AUC area of 0.9893 in this state (Fig. 9). It proves that eliminating factors with lower contribution rates can simplify the model, reduce unnecessary complexity, reduce the risk of overfitting, improve the generalization ability of the model, and reduce the dimensionality of the feature space. The feature optimization can improve the computational efficiency and training speed of the model, as well as improves stability and prediction accuracy^46^. The remaining 15 features were input into the random forest model for an operation to obtain the predicted spatial distribution of station sites in Lanzhou City. To further analyze the suitability of the completed metro station sites, they were spatially overlaid with the metro station sites predicted in this paper (Fig. 10a).

Except for the South SHEN'AN Bridge, Lanzhou City College and Matan, the predicted station sites have a good degree of conformity with the built sites in terms of spatial distribution, especially the Provincial Government, XIZHANSHIZI and XIGUAN, which have good spatial consistency, indicating that our prediction model established in this study has a high degree of confidence.

**Figure 11:**
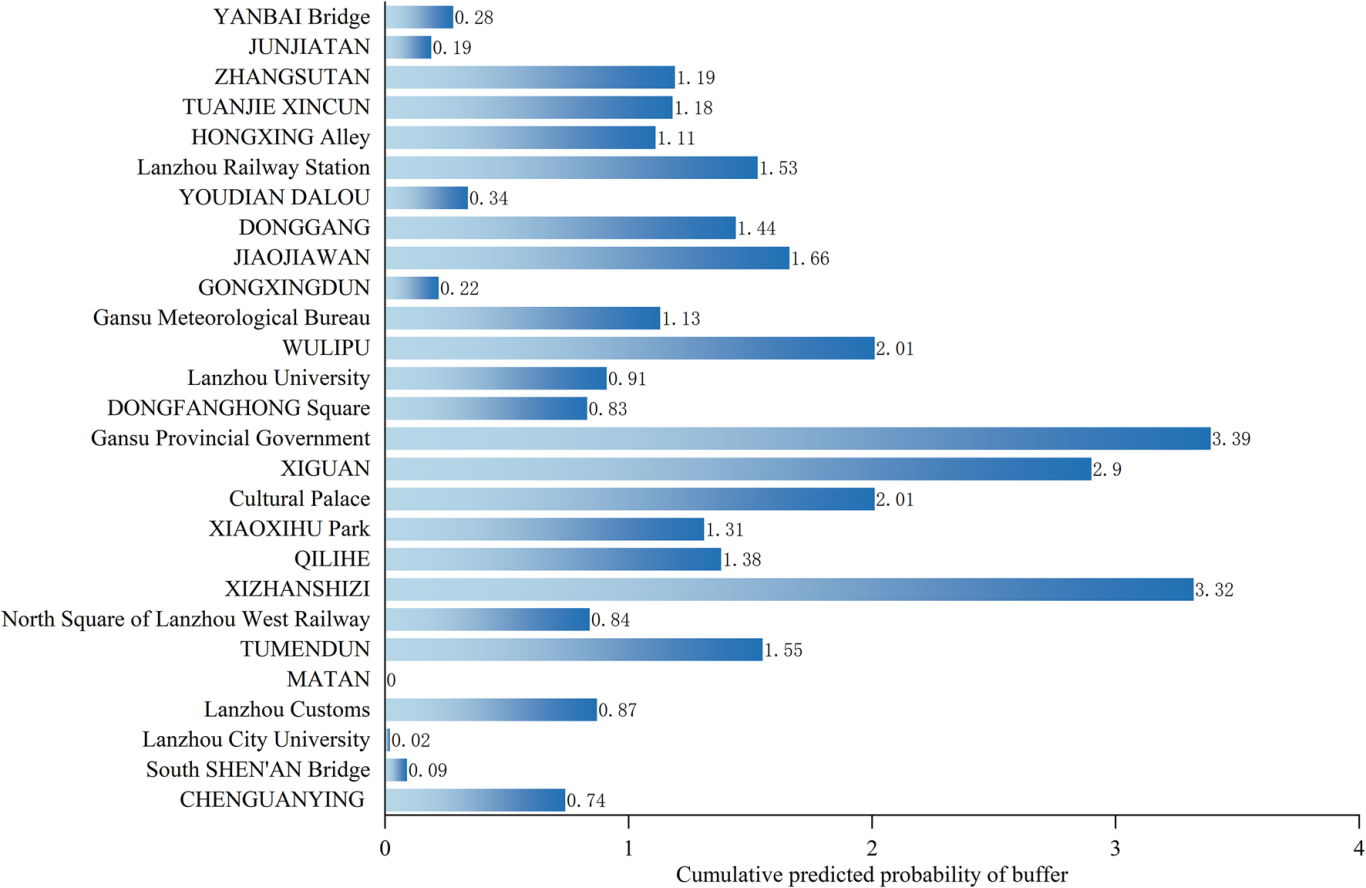
Cumulative predicted probability of the buffer zone of built subway stations.

To further analyze the relationship between the built metro station sites and the predicted sites, a buffer zone was established with a radius of 300 m (Fig. 10), and if the cumulative prediction probability of the grid falling within the buffer zone is higher, it indicates that the built metro station sites are in good agreement with the predicted sites. The cumulative prediction probability results within the buffer zone were statistically obtained (Fig. 11). The metro stations with higher cumulative predicted probability are mainly located in the Provincial Government, XIZHANSHIZI, XIGUAN, Cultural Palace and WULIPU, with cumulative predicted probability values of 3.39, 3.32, 2.9, 2.01 and 2.01 in the buffer zone respectively. They are located in areas of high pedestrian density, with high levels of economic development, transport networks and population concentrations, making them suitable locations. At the same time, the cumulative predicted probabilities of the three stations, namely MATAN, Lanzhou City College and South SHEN'AN Bridge, are relatively low, with cumulative predicted probability values of 0, 0.02 and 0.09 respectively, indicating that their suitability for location is average. Although the infrastructure construction in these areas has been basically completed, the surrounding living services and commercial facilities are still being improved, so there are some deviations between the predicted and completed stations.

**Figure 12:**
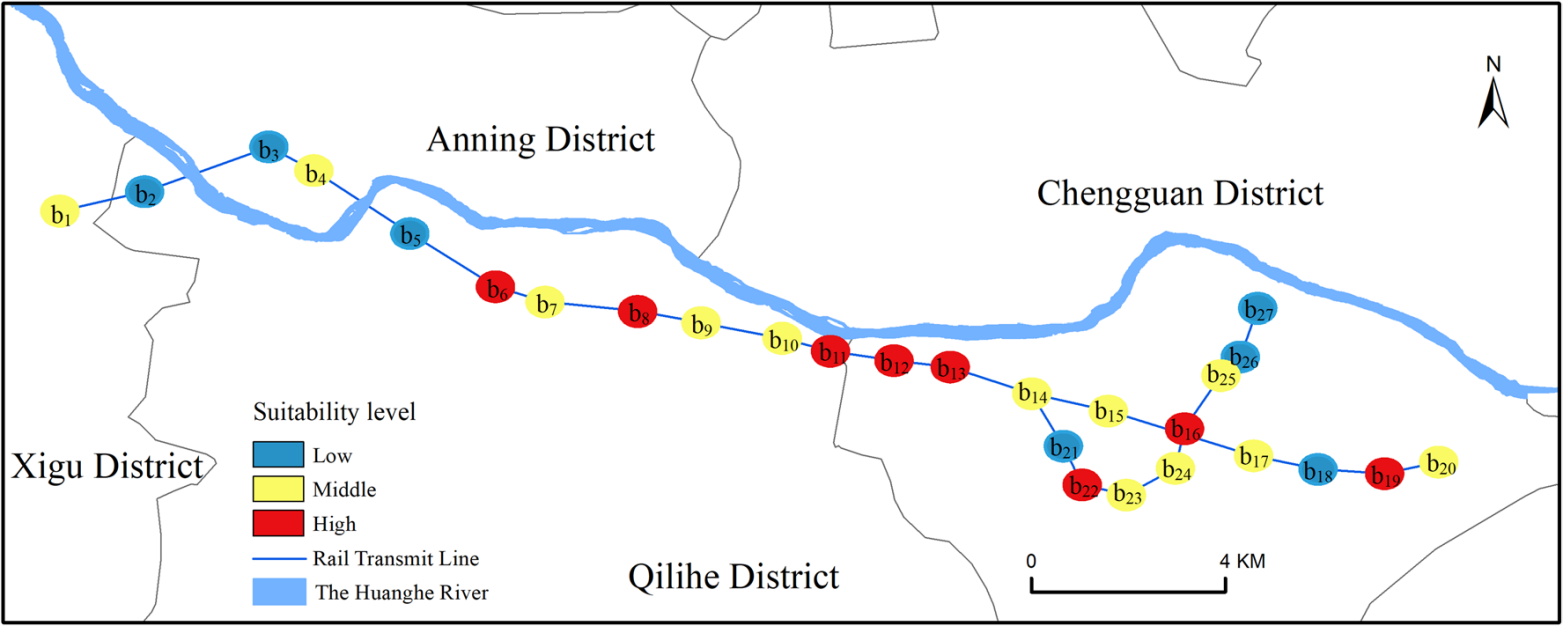
Suitability ratings of completed subway stations.

Based on the cumulative predicted probability values of the buffer zone and combined with the field survey, the suitability of the built subway stations in the study area was classified into high, medium and low according to the cumulative predicted probability values (Fig. 12). The stations with cumulative predicted probability values between 0.5 and 1.5 are of medium suitability, including 12 stations, namely CHENGUANYING, Lanzhou Customs, Lanzhou West Railway, QILIHE, XIAOXIHU Park, DONGFANGHONG Square, Lanzhou University, Gansu Meteorological Bureau, DONGGANG, HONGXING Alley, TUANJIEXINCUN and ZHANGSUTAN. The metro stations with cumulative predicted probability values greater than 1.5 are of higher suitability, including eight subway stations, i.e., TUMENDUN, XIZHANSHIZI, Culture Palace, Provincial Government, XIGUANSHIZI, Lanzhou Railway Station, WULIPU and JIAOJIAWAN.

## Discussion

### The optimization of feature factors on site suitability evaluation

As a long and typical river valley city, Lanzhou is limited by the geographical environment, such as narrow north‒south and long east‒west, and its transportation lines spread out in the east‒west are very congested. To alleviate traffic pressure and speed traffic efficiency and improve the environment, planning a subway that crosses from east to west in the city can basically meet the needs of people's travel. Therefore, it is particularly important to set up reasonable subway station sites. To comprehensively consider the feature factors for selecting and optimizing subway station sites, 19 feature factors closely related to the subway station sites in the study area were selected from four categories: natural environment, transportation location, social economy, and urban functional structure. A database of characteristic factors for subway station site selection was constructed. However, it is not that the more feature factors selected, the better the model built. Instead, the selected feature factors should be optimized in combination with the actual situation in the study area, which is conducive to improving the prediction accuracy. Based on the analysis of these feature factors, the contribution probability of each feature factor to the model was obtained based on the MDG method, and the importance ranking of each factor was obtained. It can be seen that the feature factors such as transportation facilities, companies and population density are in the top three positions, which is consistent with the actual situation of the study area. All metro station sites should be located in areas that are connected to public transportation facilities and can facilitate passenger passage and reduce commuting times. At the same time, it can be seen that the factors with lower contribution probabilities are land use, slope, and elevation. Metro stations are mainly built in urban underground spaces, which have low spatial heterogeneity with these factors. Therefore, in this study, the model was constructed after removing the feature factors with lower contribution probability, its test accuracy of tenfold cross-validation and out-of-bag data (OOB) was 89.62% and 91.285%, respectively, and the AUC area was 0.9823. This shows that the feature factors selected in this study can basically reflect the actual subway station sites in the study area, and the established model is more reliable.

### The spatial consistency between predicted sites and built sites

The purpose of this study is to provide a reference for planning station sites of new subway lines based on evaluating the spatial location suitability of the built subway station sites. Therefore, the method is designed as follows: taking the built station site as the center, we create buffers with a radius of 300 m and calculate the cumulative prediction probability of falling into the buffer grid. Then, we analyze their suitability according to the cumulative prediction probability value. Statistics found that the subway station sites with a large cumulative probability include five stations such as Provincial Government, XIZHANSHIZI, XIGUANSHIZI, Cultural Palace and WULIPU. The field investigation found that these five stations are also located on the main roads of the central urban area of the study area, adjacent to a few large enterprises and institutions, commercial centers and areas with high crowd density. In these places, the economic development level, traffic network and population concentration degree are relatively higher, so the suitability of these stations is better. At the same time, it can be seen that the cumulative prediction probabilities of the three subway stations, MATAN, Lanzhou City College and South SHEN'AN Bridge, are relatively low because these three subway stations are distributed on both sides of the Yellow River, and their surrounding living services and commercial facilities are not perfect. On the other hand, it hints at the direction of future urban development. In summary, except for MATAN, Lanzhou City College and South SHEN'AN Bridge, the built sites and predicted sites have good consistency in spatial distribution, which not only shows that the feature factors selected in this study and the model constructed are theoretically reliable but also further indicates that the conclusions of this study can basically reflect the actual situation in the study area, and our method can provide a theoretical reference for subway station site planning in this area.

### Future work for optimizing of subway station sites

In this study, only the feature factors related to subway station sites were selected from the natural environment, transportation location, socioeconomic and urban functional structure. The optimization of feature factors is based on their contribution probability to the model, and then we constructed the model to explore the suitability of the built subway station sites. However, the correlation between feature factors is still discussed because too many feature factors do not play a positive role in improving prediction accuracy^47–49^. Therefore, two challenges remain when conducting future studies on the suitability evaluation of rail transit sites. One challenge is how to select the right feature factors with low correlation and strong independence. Some studies used models such as the Pearson correlation coefficient to analyze the correlation between feature factors. However, these models are limited by their own conditions of application. Another challenge is the complexity of selecting subway station sites. It can be interfered with by a variety of factors, such as local government rules, competitors, land prices, and construction feasibility. Therefore, it is important to use comprehensive feature factors and up-to-date information data as well as feasible methods to study the site optimization of subway stations.

## Conclusions

In the long-stripped, typical valley-type city of Lanzhou, the feature factors closely related to the subway station site in the study area were selected from the natural environment, transportation location, socioeconomic and urban functional structure. We constructed the model after removing the feature factors with low contribution probability. Our results show that the feature factors selected in this study can basically reflect the actual situation of subway station sites in the study area. The superposition analysis of the predicted sites and the built sites shows that except for MATAN, Lanzhou City College and South SHEN'AN Bridge, the spatial distribution of the others has good consistency with our predicted sites, which is consistent with the actual situation in the study area. Our conclusions can provide a suitability evaluation of the rail transit sites in Lanzhou and provide a theoretical reference for site planning and optimization of new rail transit lines in this area.

## Data Availability

Traffic facility data was obtained from https://lbs.amap.com/ (Selected data for 2022); Accommodation services data was downloaded from https://lbs.amap.com/ (Selected data for 2022); Business residential data from https://lbs.amap.com/ (Selected data for 2022); Corporate enterprise data from https://lbs.amap.com/ (Selected data for 2022); Financial and insurance services data from https://lbs.amap.com/ (Selected data for 2022); Food and beverage service data from https://lbs.amap.com/ (Selected data for 2022); Government agencies data from https://lbs.amap.com/ (Selected data for 2022); Healthcare services data from https://lbs.amap.com/ (Selected data for 2022); Life Services data from https://lbs.amap.com/ (Selected data for 2022); Scenic spots data from https://lbs.amap.com/ (Selected data for 2022); Science and education culture data from https://lbs.amap.com/ (Selected data for 2022); Shopping services data from https://lbs.amap.com/ (Selected data for 2022); Sports and leisure services data from https://lbs.amap.com/ (Selected data for 2022); Transportation facilities data from https://lbs.amap.com/ (Selected data for 2022); population counts from https://www.worldpop.org/ (Selected data for 2022); Nighttime lighting data from http://59.175.109.173:8888/index.html (Data from the Luojia-1 2021 satellite launched by Wuhan University); DEM (Digital Elevation Model, DEM) from https://earthdatanasa.gov/ (Data published on the official website of NASAEARTH DATA in 2019); land use data from http://data.casearth.cn/sdo/detail/5fbc7904819aeclea2dd7061 (Use data to 2020); road network from https://www.openstreetmap.org (Data from the official OpenStreetMap website for 2022).
